# Construction of a Novel Ferroptosis-Related Gene Signature of Atherosclerosis

**DOI:** 10.3389/fcell.2021.800833

**Published:** 2022-01-05

**Authors:** Tucheng Huang, Kangjie Wang, Yuewei Li, Yanchen Ye, Yangxin Chen, Jingfeng Wang, Chen Yao

**Affiliations:** ^1^ Department of Cardiology, Sun Yat-sen Memorial Hospital, Sun Yat-sen University, Guangzhou, China; ^2^ Laboratory of Cardiac Electrophysiology and Arrhythmia in Guangdong Province, Guangzhou, China; ^3^ Division of Vascular Surgery, The First Affiliated Hospital, Sun Yat-sen University, Guangzhou, China; ^4^ National-Guangdong Joint Engineering Laboratory for Diagnosis and Treatment of Vascular Diseases, First Affiliated Hospital, Sun Yat-sen University, Guangzhou, China; ^5^ Department of Respiratory Medicine, Sun Yat-Sen Memorial Hospital, Sun Yat-sen University, Guangzhou, China

**Keywords:** atherosclerosis, ferroptosis, carotid artery, ceRNA, bioinformatics

## Abstract

Atheroclerosis refers to a chronic inflammatory disease featured by the accumulation of fibrofatty lesions in the intima of arteries. Cardiovasular events associated with atherosclerosis remain the major causes of mortality worldwide. Recent studies have indicated that ferroptosis, a novel programmed cell death, might participate in the process of atherosclerosis. However, the ferroptosis landscape is still not clear. In this study, 59 genes associated with ferroptosis were ultimately identified in atherosclerosis in the intima. Gene ontology (GO) and Kyoto Encyclopedia of Genes and Genomes (KEGG) pathway enrichment analyses were performed for functional annotation. Through the construction of protein–protein interaction (PPI) network, five hub genes (*TP53*, *MAPK1*, *STAT3*, *HMOX1*, and *PTGS2*) were then validated histologically. The competing endogenous RNA (ceRNA) network of hub genes was ultimately constructed to explore the regulatory mechanism between lncRNAs, miRNAs, and hub genes. The findings provide more insights into the ferroptosis landscape and, potentially, the therapeutic targets of atherosclerosis.

## Introduction

Coronary artery disease (CAD) and stroke, caused by atherosclerosis (AS), are considered as types of chronic inflammatory arterial diseases characterized by the accumulation of lipids and the formation of atherosclerotic plaques (Benjamin et al., 2017; Libby et al., 2019). Endothelial dysfunction and low-density lipoprotein cholesterol (LDL) infiltration into the subendothelial layer of arteries initiated atherogenesis (Peluso et al., 2012; Förstermann et al., 2017; Kattoor et al., 2017). Lipid peroxidation played a significant part in the pathogenesis of AS (Gisterå and Hansson, 2017).

Ferroptosis is a form of programmed cell death definitely modulated by iron-dependent lethal lipid peroxidation. Morphologically, ferroptosis is characterized by vanished mitochondria cristae and condensed and ruptured mitochondrial membranes (Mou et al., 2019). Iron overload or the inactivation of glutathione peroxidase 4 (*GPX4*) promoted reactive oxygen species (ROS), which hastened lipid peroxidation, eventually leading to ferroptosis (Tang et al., 2021). ROS was implicated in endothelial dysfunction, which was the initiating link of AS (Maiocchi et al., 2021). Recent studies have elucidated the status of ferroptosis in AS (Bai et al., 2020; Stockwell et al., 2020; Zhou et al., 2021). Ferrostatin-1 (Fer-1) alleviated the atherosclerotic lesion in high-fat diet (HFD)-fed ApoE^−/−^ mice, probably *via* attenuating the endothelial dysfunction induced by oxidation-modified LDL (ox-LDL) (Bai et al., 2020). Prostaglandin-endoperoxide synthase 2 (*PTGS2*), a ferroptosis-related protein, positively correlated with the severity of AS (Zhou et al., 2021). However, how ferroptosis regulates the progress of AS still requires further investigation.

In the present study, datasets were downloaded from the Gene Expression Omnibus (GEO). Afterward, bioinformatics analyses were performed to select differentially expressed genes (DEGs). Combined with ferroptosis-related genes (FRGs), five hub genes—*TP53*, *MAPK1*, *STAT3*, *HMOX1*, and *PTGS2*—were ultimately screened and validated histologically.

## Materials and Methods

### Data Collection and Acquisition of Ferroptosis-Related Gene

The RNA expression data were collected from the GEO (http://www.ncbi.nlm.nih.gov/geo/) database with series numbers GSE97210, GSE125771, GSE41571, and GSE28829. FRGs that drive, suppress, or mark ferroptosis were retrieved from the public FerrDb database (http://www.zhounan.org/ferrdb). After removing repetitive genes, 149 FRGs that were validated by experiments were eventually obtained for subsequent analyses.

### Identification of Differentially Expressed Ferroptosis-Related Genes

The “AnnoProbe” package was employed in the re-annotation of the series matrix. The “limma” package in R software was utilized to calibrate the microarray data and identify the DEGs between the atherosclerotic plaques and normal arterial intimae. The messenger RNAs (mRNAs) and long non-coding RNAs (lncRNAs) that meet the defined criteria, |log2FC| ≥ 1 and adjusted *p* < 0.05, were considered as DEGs and differentially expressed lncRNAs (DElncRNAs), respectively. Thereafter, the intersecting genes between DEGs and FRGs were defined as the differentially expressed ferroptosis-related genes (DE-FRGs). The DEGs and DElncRNAs were displayed in volcano plots based on the “ggplot2” package. The number of DE-FRGs was shown in a Venn diagram using the “Venndiagram” package. The expressions of DE-FRGs were visualized in a heatmap with the “ggplot2” package.

### Gene Set Enrichment Analysis in Atherosclerotic Plaque

Gene set enrichment analysis (GSEA) was employed to detect the related signaling pathways in atherosclerotic plaque progression, which was performed using the OmicStudio online tool (http://www.omicstudio.cn/tool). The significant gene sets that conform to the nominal (NOM) *p*-value <0.05 and false discovery rate (FDR) <25% were shown.

### Functional Annotation and Pathway Enrichment of DEGs and DE-FGRs

Gene Ontology (GO) biological process and Kyoto Encyclopedia of Genes and Genomes (KEGG) annotation were performed using the Metascape website (http://metascape.org). Biological process and KEGG pathway enrichment analyses using the “ClusterProfiler” package were performed to obtain insights into the potential functions of the DEGs and DE-FRGs. The top 20 results were shown in the enrichment scatter plots.

### Construction of Protein–Protein Interaction Network of DE-FRGs

The STRING database (http://string-db.org/) was employed to analyze the interactions of the distinct DE-FRGs. Cytoscape software 3.8.1 (http://cytoscape.org/) was then utilized to construct and visualize the protein–protein interaction (PPI) network. The molecular complexes were examined using the MCODE algorithm. The top 5 genes of the PPI network were defined as the hub genes, which were calculated based on the maximum neighborhood component (MNC), degree, and edge percolated component (EPC) algorithms by utilizing the cytoHubba plug-in.

### Identification of the Correlation of Hub FRGs and Mitochondrial Function-Related Genes

A total of 1,262 genes related to mitochondrial function were obtained from the online database Integrated Mitochondrial Protein Index (IMPI; https://mitominer.mrc-mbu.cam.ac.uk/release-4.0/impi.do). The intersecting genes of the DEGs and mitochondrial function-related genes (MFRGs) were defined as the differentially expressed mitochondrial function-related genes (DE-MFRGs). Pearson’s correlation analysis between the hub genes and DE-MFRGs was performed utilizing the “stats” package. All results were displayed in a heatmap.

### Validation of Hub Gene Expression of Atherosclerotic Plaque Datasets

The three microarray datasets of atherosclerotic plaques (GSE28829: *n* = 29, advanced *vs*. early plaques; GSE125771: *n* = 16, high *vs*. low calcified plaques; and GSE41571: *n* = 11, ruptured *vs*. stable plaques) that were retrieved from the GEO database were used to verify the expressions of the hub genes. The “limma” package was also applied to identify the DEGs with thresholds of |log2FC| ≥ 1 and adjusted *p* < 0.05. The results were visualized in volcano plots and the hub genes were marked.

### Atherosclerosis Animal Model Procedure

The animal experiment was reviewed and approved by the Animal Ethics Committee, which was subordinate to the First Affiliated Hospital of Sun Yat-Sen University (permit no. 2021593). Five-week-old male *Apoe* ^*−/−*^ mice (purchased from GemPharmatech Co., Ltd., Nanjing, China) were subsequently randomly divided into two groups. The mice of the control group were fed with normal chow diet for 12 weeks; the mice of the high-fat group were fed a high-fat diet for 12 weeks. All the mice were sacrificed after feeding for 12 weeks, and the heart tissues, including aortic roots, were collected.

### Hematoxylin and Eosin, Oil Red O, and Masson Staining

Hematoxylin and eosin (HE) and Oil Red O staining were conducted in accordance with the methods mentioned in a previous study (Jiang et al., 2020). The atherosclerotic lesions in the aortic valve of mice were observed with a microscope (CKX53; Olympus Optical Co., Ltd., Tokyo, Japan).

### Immunohistochemistry Staining of Hub Gene Validation

For immunohistochemistry (IHC), the heart tissues were harvested and fixed for 24 h with 10% neutral buffered formalin before the optimal cutting temperature (OCT) embedding procedure. Then, the embedded blocks were processed as 5-μm cryosections that were fixed in 4% paraformaldehyde (PFA) for another 10 min. After washing with phosphate-buffered saline (PBS) three times, antigen retrieval was followed by the heat method for 3 min. The slides were washed in PBS for another three times, blocked in 5% bovine serum albumin (BSA) for 1 h at 37°C, and then incubated with *HOMX1* antibody (GB11549; Servicebio, Wuhan, China) at 1:600 dilution, *MAPK1* antibody (GB11370-1; Servicebio) at 1:200 dilution, PTGS2 antibody (GB11077-1; Servicebio) at 1:500 dilution, *TP53* antibody (10442-1-AP; Proteintech, Wuhan, China) at 1:200 dilution and *STAT3* antibody (ET1607-38; Huabio) at 1:100 dilution at room temperature for 60 min. Staining followed the IHC kit protocol of Servicebio, and the nuclei were visualized by hematoxylin staining and examined using the CKX53 microscope (Olympus Optical Co., Ltd.).

### Construction of the lncRNA–miRNA–mRNA ceRNA Network

The co-expressions of DElncRNAs and hub genes were analyzed with Pearson’s correlation. Only DElncRAN–hub gene pairs with a correlation coefficient >0.5 and *p* < 0.05 were selected. Subsequently, the ENCORI dataset (starbase.sysu.edu.cn) was employed to determine potentially interacting lncRNA–microRNA (miRNA) pairs. The miRanda, TargetSacan, and ENCORI databases were chosen to identify miRNA–mRNA pairs. Finally, lncRNA–miRNA–mRNA networks, which comprised five hub FRGs, were ultimately constructed.

## Results

### Identification of DE-FRGs and DElncRNAs in Atherosclerotic Plaques

**FIGURE 1:**
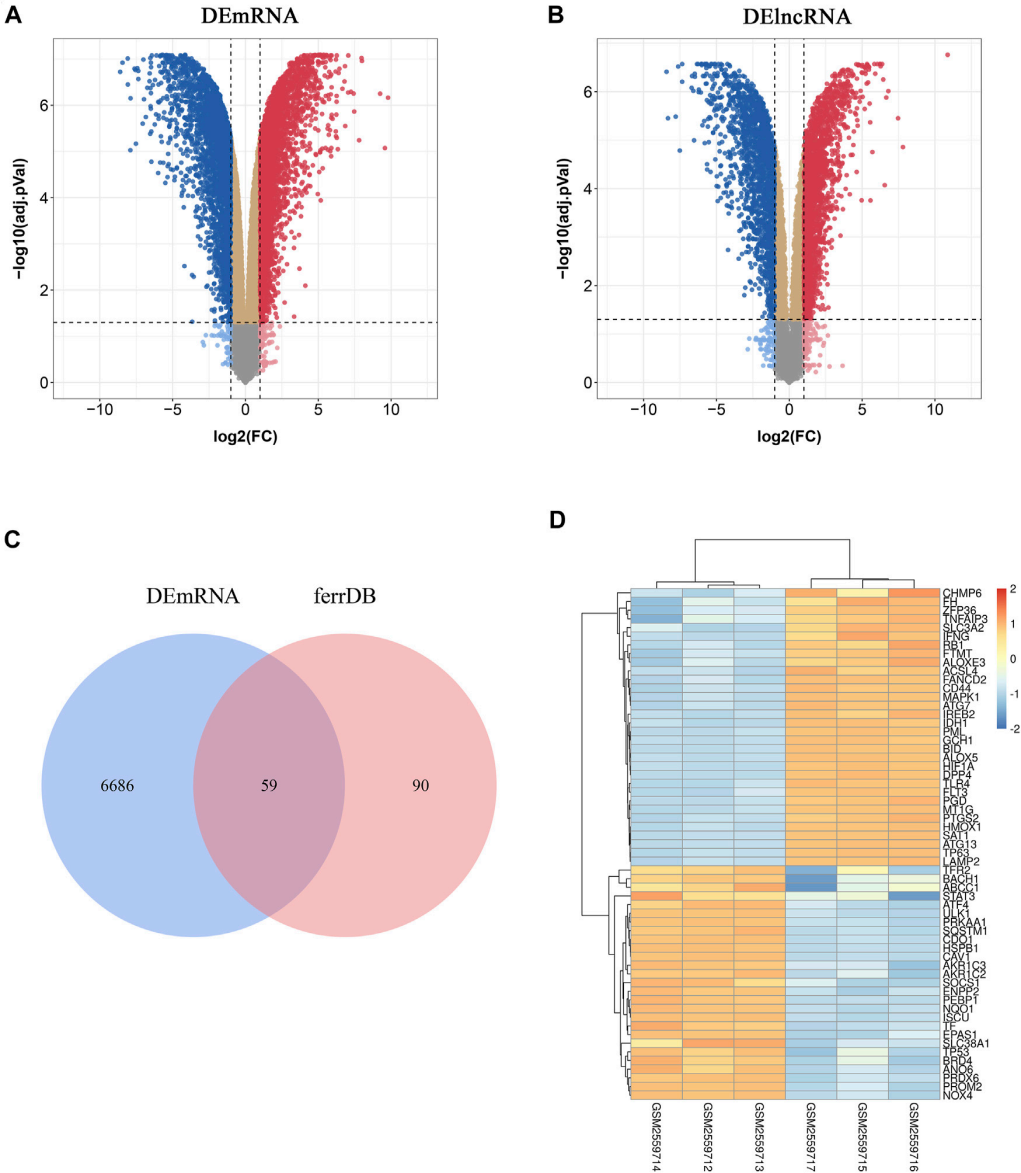
Identification of differentially expressed ferroptosis-related genes (DE-FRGs) and differentially expressed long non-coding RNAs (DElncRNAs). **(A**,**B)** Volcano plots displaying significantly differentially expressed genes **(A)** and lncRNAs **(B)** in atherosclerotic plaques. *Red dots* represent the upregulated genes and *blue dots* denote the downregulated genes, with thresholds of |log2FC| ≥ 1 and adjusted *p* < 0.05. **(C)** Venn diagram displaying the DE-FRGs. **(D)** Heatmap displaying the expressions of the 59 DE-FRGs in atherosclerotic plaques. *Red bricks* indicate the more highly expressed FRGS and *blue bricks* indicate lower expression.

To investigate FRGs differentially expressed in atherosclerotic plaques, 149 FRGs were extracted from FerrDb, a database for regulators, markers, and diseases associated with ferroptosis. Through differential expression analysis of GSE97210, 4,017 lncRNAs and 6,754 genes were significantly differentially expressed in atherosclerotic plaques compared with normal intimae, with thresholds of |log2FC| ≥ 1 and adjusted *p* < 0.05 (Figures 1A, B). After taking the intersection of DEGs and FRGs, a total of 59 FRGs expressed differentially were defined as DE-FRGs (Figure 1C). The expressions of the 59 DE-FRGs were visualized in a heatmap (Figure 1D).

### Gene Set Enrichment Analysis

**FIGURE 2:**
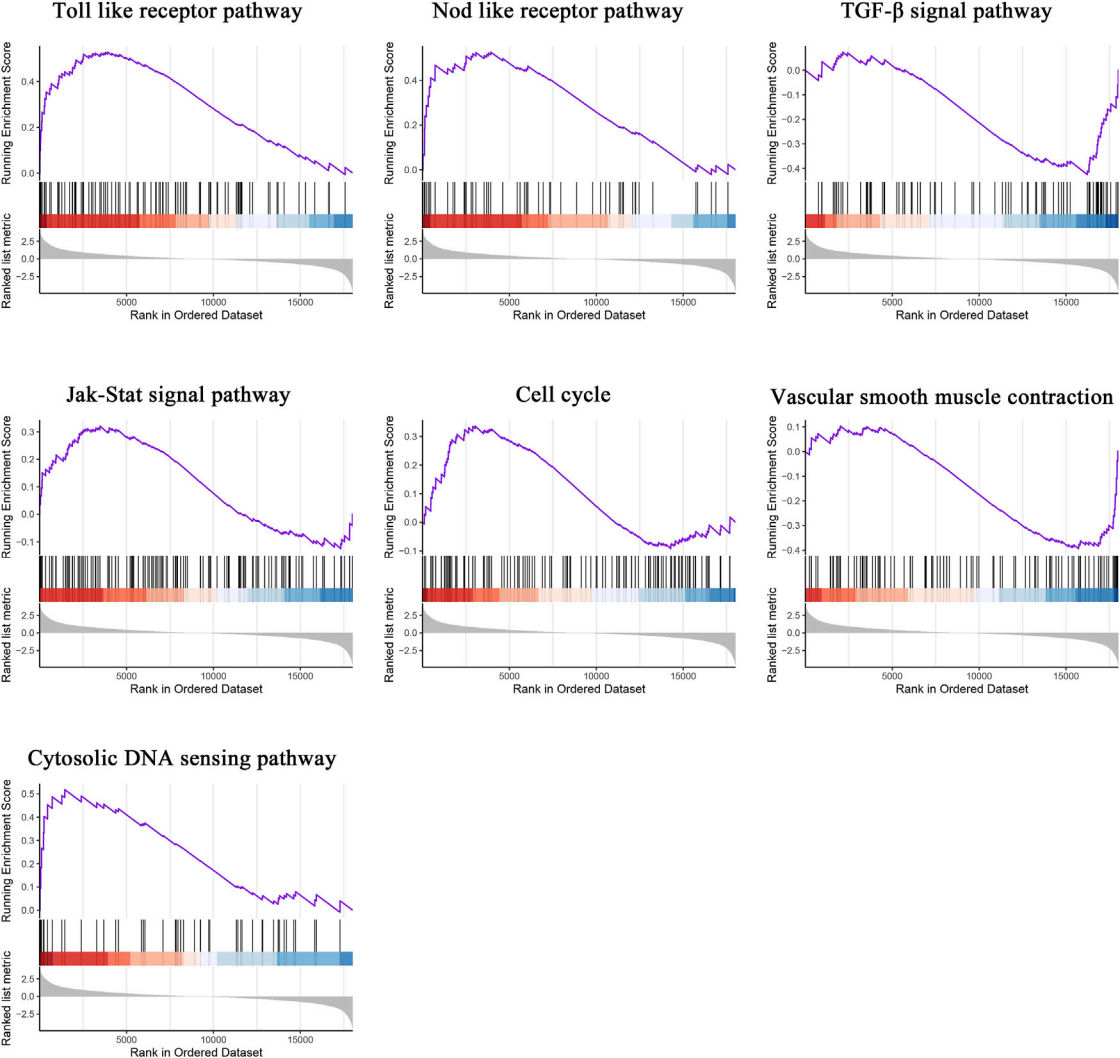
Gene set enrichment analysis. GSEA in atherosclerotic plaques. NOM *p* < 0.05, FDR < 25%.

To compare the distinct pathways between the two groups, GSEA was subsequently conducted. The Toll-like receptor pathway, NOD-like receptor pathway, transforming growth factor beta (TGF-β) signal pathway, Janus kinase (JAK)/signal transducer and activator of transcription (STAT) signal pathway, cell cycle, vascular smooth muscle contraction, and cytosolic DNA sensing pathway were significantly enriched in atherosclerotic plaques (Figure 2).

### Functional Enrichment Analysis of DEGs and DE-FRGs

**FIGURE 3:**
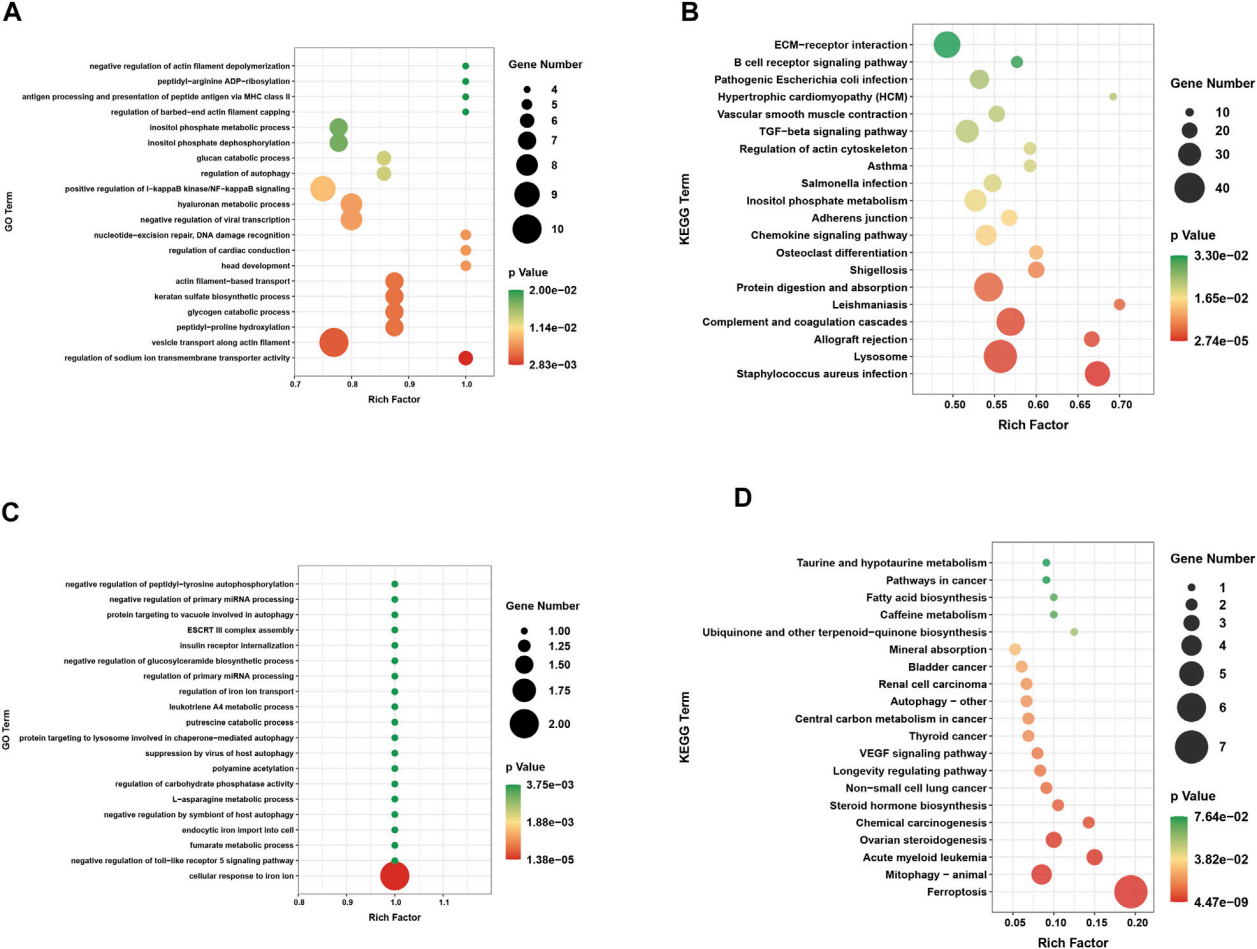
Functional annotation of DEGs and DE-FRGs. **(A)** GO enrichment analysis of biological processes enriched by DEGs. **(B)** Annotation of pathway enriched by DEGs. **(C)** GO enrichment analysis of biological processes enriched by DE-FRGs. **(D)** KEGG pathway enrichment analysis enriched by DE-FRGs.

Based on GO and KEGG pathway analyses, exploration of the potential biological functions and pathways of DEGs was conducted for the two groups. The top 20 results were shown in the enrichment scatter plots. Based on GO analysis, the DEGs were significantly enriched in related processes such as positive regulation of IκB kinase/NF-κB signaling and regulation of autophagy (Figure 3A). Investigation of the KEGG pathway analysis primarily suggested that these DEGs were involved in TGF-β signaling pathway, chemokine signaling pathway, and vascular smooth muscle contraction (Figure 3B). In addition, GO analysis was performed on DE-FRGs, for which the results indicated that the DE-FRGs were involved in iron transport, autophagy, and negative regulation of the Toll-like pathway (Figure 3C). As expected, these DE-FRGs were significantly enriched in ferroptosis, mitophagy, and autophagy, as indicated by the KEGG pathway analysis (Figure 3D).

### Protein–Protein Interaction Network Construction and Visualization

**FIGURE 4:**
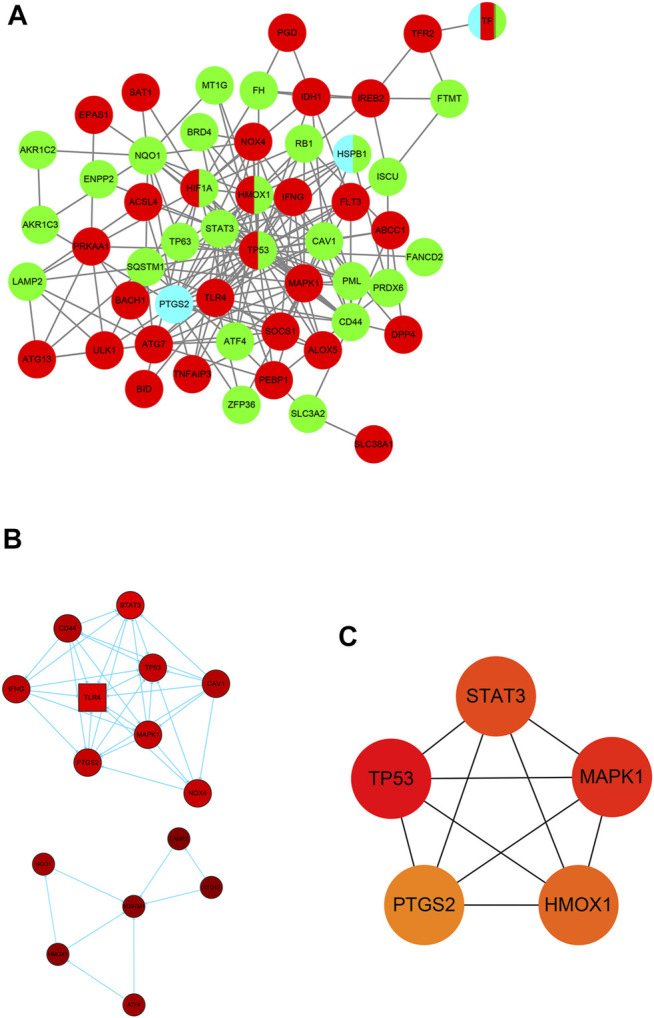
Identification of five hub ferroptosis-related genes (FRGs). **(A)** Protein–protein interaction (PPI) network of 59 differentially expressed FRGs (DE-FRGs). *Red* indicates the driver of ferroptosis, *light blue* indicates the marker of ferroptosis, and *green* indicates the suppressor of ferroptosis. **(B)** Two clusters of the PPI network. **(C)** Hub genes calculated using the maximum neighborhood component (MNC), degree, and edge percolated component (EPC) algorithms.

For the purpose of exploring the interactions between each DE-FRG, all DE-FRGs were submitted to the STING database, which is well known for PPI. The PPI network was established and visualized using Cytoscape 3.8.1. After removing the isolated DE-FRGs, the PPI networks of DE-FRGs were displayed including 54 nodes and 190 edges (Figure 4A). According to the scores calculated using the MCODE algorithm, the PPI networks were divided into two clusters (Figure 4B). The first cluster was composed of nine genes (*TP53*, *CD44*, *STAT3*, *CAV1*, *TLR4*, *IFNG*, *MAPK1*, and *PTGS2*), whereas the second cluster consisted of six genes (*LAMP2*, *NQO1*, *SQSTM1*, *ATG13*, *HMOX1*, and *ATF4*). Subsequently, the top 5 intersecting genes analyzed based on the MNC, degree, and EPC algorithms were selected as the hub genes, which included *TP53*, *MAPK1*, *STAT3*, *HMOX1*, and *PTGS2* (Figure 4C).

### Identification of the Relationship Between Hub Genes and Mitochondrial Function

Since ferroptosis is always accompanied by mitochondrial dysfunction (Battaglia et al., 2020; Otasevic et al., 2021), the relationship between hub genes and MFRGs was analyzed. Among the 1,262 MFRGs, 505 genes were eventually classified as DE-MFRGs (Supplementary Figure S1A). A total of 210 DE-MFRGs were upregulated and 295 were downregulated. Pearson’s correlation analysis showed that most DE-MFRGs were highly correlated with the hub FRGs, whether positive or negative (|*r*| ≥ 0.5, *p* < 0.05) (Supplementary Figure S1B).

### Validation of Hub Gene Expression in Atherosclerotic Plaque

**FIGURE 5:**
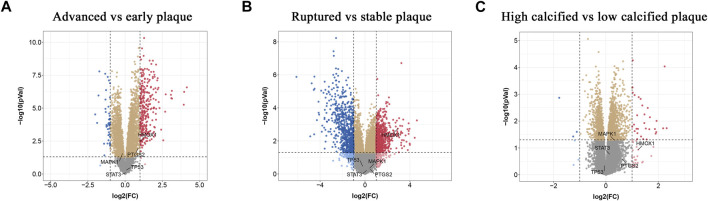
Validation of the expressions of hub ferroptosis-related genes (FRGs) in advanced, ruptured, and calcified atherosclerotic plaques. **(A–C)** Volcano plots displaying the expressions of hub genes in advanced, ruptured, and highly calcified plaques. *Red dots* indicate high relative expression and *blue dots* denote low relative expression.

**FIGURE 6:**
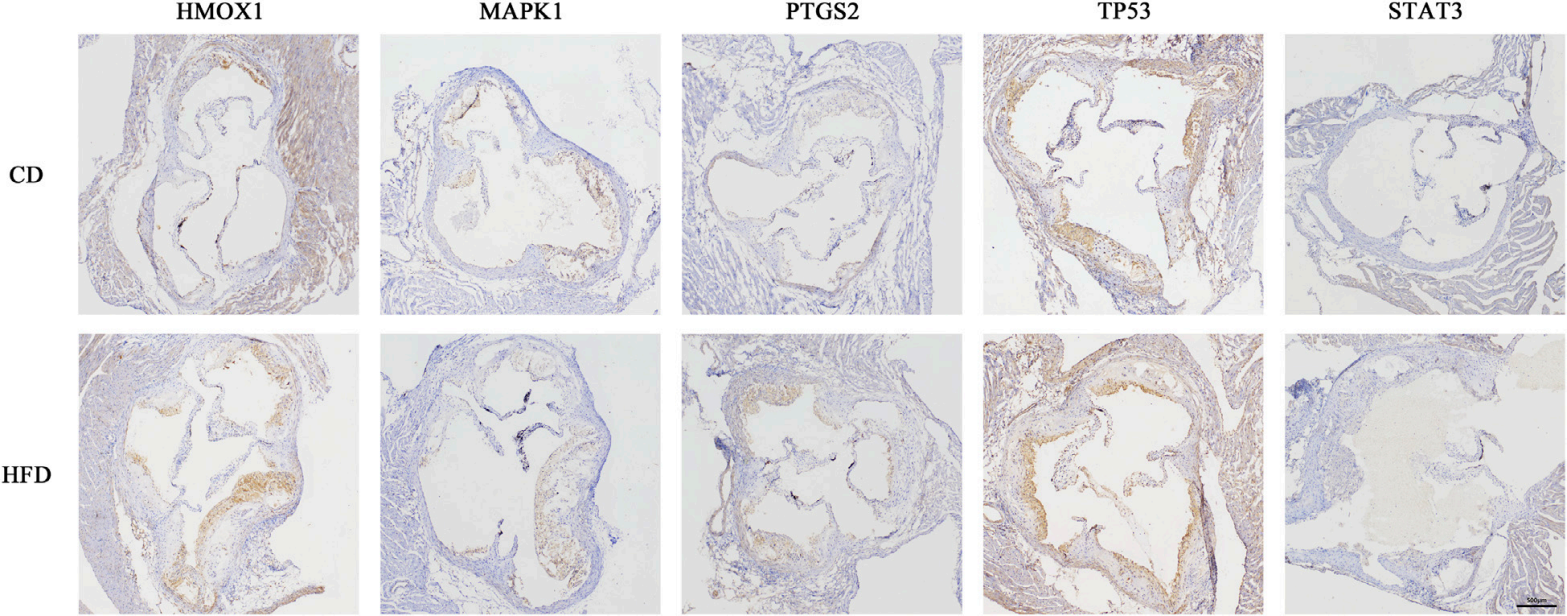
Five hub genes expressed in atherosclerotic plaques of *Apoe* ^*−/−*^ mice. Immunohistochemical staining of the aortic valve from *Apoe* ^*−/−*^ mice using *HMOX1, MAPK1, PTGS2, TP53,* and *STAT3* antibodies.

To find out whether the hub genes were differentially expressed in other types of atherosclerotic plaques, another three microarray datasets (GSE28829, GSE125771, and GSE41571) were chosen for analysis. A total of 320 DEGs were identified between early and advanced plaques, 34 between low and highly calcified plaques, and 1,431 between ruptured and stable plaques (Figures 5A–C). Only *HMOX1* among the 5 hub genes was consistently upregulated in advanced and ruptured atherosclerotic plaques, which suggested that a higher level of *HMOX1* expression could predict more serious kinds of atherosclerotic plaques. *HMOX1* was also highly expressed in highly calcified atherosclerotic plaques, although there was no statistical significance. To determine the expression of the five top-ranking hub genes in atherosclerotic plaques, a preclinical model of AS was generated. Compared with the control, more obvious atherosclerotic plaques and lipid accumulation were observed in mice fed the Western diet. Likewise, atherosclerotic plaques exhibited apparent fibrous caps, as revealed by Masson’s stain (Supplementary Figure S2). As expected, *HMOX1* and *PTGS2* were highly expressed in atherosclerotic plaques induced by the Western diet (Figure 6). However, no differences in *MAPK1*, *TP53* and *STAT3* were seen between the two groups.

### Construction of the lncRNA–miRNA–mRNA ceRNA Network

**FIGURE 7:**
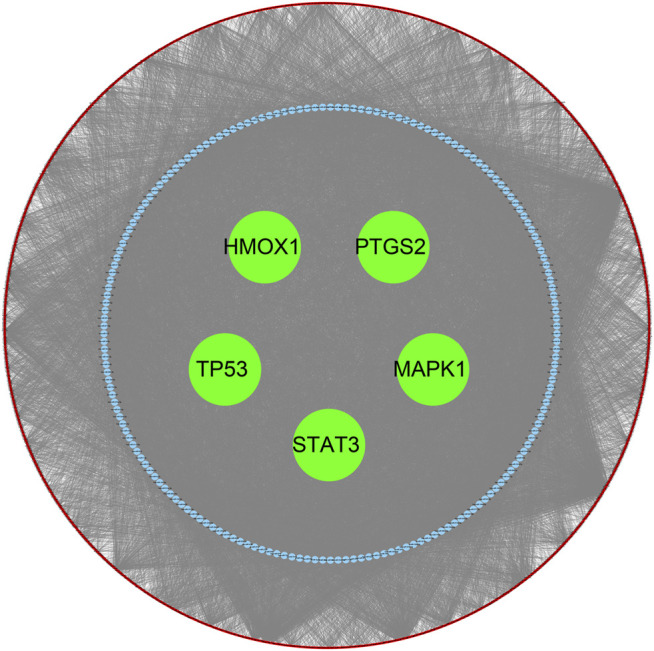
Construction of the ceRNA Network. *Red nodes* indicated the predicted lncRNAs. *Blue nodes* indicated the predicted miRNAs. *Green nodes* indicated the hub genes.

Based on the competitive endogenous RNA hypothesis, lncRNA–miRNA–mRNA competing endogenous RNA (ceRNA) networks were constructed to explore the functions of lncRNAs acting as miRNA sponges in atherosclerotic plaques (Figure 7). The co-expressed upregulated lncRNA and hub FRG pairs were integrated into the upregulated ceRNA network with the predicted miRNAs. This ceRNA network contained 1,439 lncRNA nodes, 184 miRNA nodes, 5 hub gene nodes, and 20,292 edges.

## Discussion

In this study, we mainly concentrated on the role of ferroptosis in the development of AS. A chronic and progressive disease of the arteries, AS is characterized by the accumulation of lipid and/or fibrous composition in the intima of arteries. Once the lipid-rich plaques rupture, a stroke or heart attack might occur, which are still the major causes of mortality worldwide (Benjamin et al., 2017). In the initial stage of AS, LDL deposition into the intima was modified by the oxidation (ox-LDL), which exhibited immunogenic and pro-inflammatory properties (Tabas et al., 2007; Tabas and Bornfeldt, 2016; Bai et al., 2020; Stockwell et al., 2020; Zhou et al., 2021). Ferroptosis was induced by excessive iron and lethal lipid peroxidation (Bai et al., 2020; Stockwell et al., 2020; Zhou et al., 2021). Excessive iron accelerated the production of ROS, which led to lipid peroxidation *via* Fenton reaction, which is necessary for ferroptosis (Pratt et al., 2011; Dev and Babitt, 2017; Stockwell et al., 2017). Several studies have demonstrated that iron overload bolstered the development of AS, and a low-iron diet or the administration of iron chelators reduced the severity of AS in preclinical models (Zhang et al., 2010; Hu et al., 2019; Cai et al., 2020; Vinchi et al., 2020). Consistently, recent studies have highlighted the importance of ferroptosis in AS. *In vitro*, ox-LDL triggered the ferroptosis of human umbilical vein endothelial cells (HUVECs) with elevated ROS generation and impaired viability, which was rescued by the activation of *PDSS2*/*Nrf2* signaling (Yang et al., 2021). In addition, in a mouse model of AS, suppression of ferroptosis by Fer-1 improved the ROS-stimulated lipid peroxidation and endothelial dysfunction, thus attenuating the atherosclerotic lesion (Bai et al., 2020).

We screened five ferroptosis-related genes—*TP53*, *MAPK1*, *STAT3*, *HMOX1*, and *PTGS2*—that are probably implicated in AS *via* bioinformatics analysis. The tumor suppressor p53 (*TP53*) is considered as a classical tumor suppressor. In response to cellular stresses such as DNA damage, hypoxia, oncogene activation, and ribosomal stress, activated *TP53* could boost cell cycle arrest, DNA damage repair, various pathways of cell death, and metabolic changes (Hafner et al., 2019). It was reported that a *TP53* mutation was involved in oncogenesis (Levine, 2020). *TP53* has been demonstrated to promote cancer ferroptosis predominantly *via* regulating *SLC7A11* expression and cystine uptake (Jiang et al., 2015). Importantly, enhancing *TP53* activity protected against AS development in HFD-fed ApoE^−/−^ mice *via* regulating smooth muscle cell proliferation and apoptosis (Mercer et al., 2005; Wu et al., 2014). *TP53* is a well-known gene for the tumor suppressor protein p53 that participates in AS (Guevara et al., 1999; Merched et al., 2003), but is also involved in lipid metabolism (Goldstein et al., 2012). Surprisingly, bioinformatics analysis indicated the relatively low expression of *TP53* in atherosclerotic plaques. IHC staining of *TP53* showed no differences between mouse atherosclerotic plaques. It seems that the expression of *TP53* could not distinguish the deteriorated plaques. This result might be due to the diversity of plaque lesions, for which further experimental evidence is needed. Heme oxygenase (*HMOX1*), a rate-limiting enzyme of heme degradation process, controls the generation of biliverdin, iron, and carbon monoxide (Stocker and Perrella, 2006). *HMOX1*, as the downstream of *Nrf2*, is involved in the maintenance of cellular homeostasis, but its role in ferroptosis is controversial in cancer cells and renal proximal tubule cells (Wang et al., 2021). The different effects may be related to different cells. In vascular cells, the role of *HMOX1* is protective in endothelial dysfunction (Nitti et al., 2020). Our study suggested that a higher level of *HMOX1* expression potentially predicts the much more serious types of atherosclerotic plaques, but independently associated with calcification. Based on this, the effect of *HMOX1* involved in ferroptosis and AS *in vitro* was investigated to further explore the mechanism responsible. *MAPK1* encoded mitogen-associated protein kinase 1 (*MAPK1*), a component of the mitogen-activated protein kinase (MAPK)/extracellular signal-regulated kinase (ERK) signaling pathway, which stimulated ferroptosis *via* increasing ROS production (Liu et al., 2021; Su et al., 2019). The downregulation of the lncRNA *MALAT1* could significantly improve the cardiac function in acute myocardial infarction and hypoxia by inhibiting the ERK/MAPK pathway (Fan et al., 2019). It is known that the *JAK2*/*STAT3* pathway is involved in AS (Wang et al., 2018) and also associated with ferroptosis (Yang et al., 2020). In *Jak2* mice, hematopoietic *Jak2* ^*VF*^ expression contributed to early lesion formation and increased complexity in advanced AS, which promoted the accumulation of iron in plaques and increased necrotic core formation (Wang et al., 2018). Furthermore, Yang et al. found that Auranofin, an anti-inflammatory drug used to treat rheumatoid arthritis, mitigates systemic iron overload and induces ferroptosis (Yang et al., 2020). *PTGS2* upregulation was suggested to be a downstream marker of ferroptosis, and it was confirmed that *PTGS2* is a hub gene of ferroptosis in human coronary artery AS (Zhou et al., 2021). A correlation was shown between the expressions of *PTGS2* and *ACSL4*, and *caspase-1* and *NLRP3(12)*. Although functional roles of FRGs were implied in our study, the involved signaling pathways and interaction networks within lncRNAs should be clarified and validated in further research.

Iron bioresorbable coronary scaffold (IBS) system was enrolled in a randomized phase III clinical trial on stent implantation in CAD patients. The preclinical study showed a relatively higher fibrin score in the IBS group, which means a higher risk of thrombogenicity than permanent cobalt–chromium alloy and durable polymer (Zheng et al., 2019). The high fibrin score and corrosion of stents might be related to ferroptosis and iron overload-regulated cell death. There was no evidence of iron bioresorbable stents promoting AS, but the stent was implanted in porcine non-atherosclerotic coronary arteries. This needs to be confirmed by further research.

In summary, we identified five ferroptosis-related genes—*TP53*, *MAPK1*, *STAT3*, *HMOX1*, and *PTGS2*—in the development of AS. However, the studies on ferroptosis and AS are still at an infant stage. More studies will reveal the limited molecular mechanism of ferroptosis, thus providing more evidence for ferroptosis in the prevention and treatment of AS. This study provides new insights into ferroptosis and AS.

## Data Availability

The original contributions presented in the study are included in the article/Supplementary Material. Further inquiries can be directed to the corresponding authors.
