# Supplementary material for: Construction of a Novel Ferroptosis-Related Gene Signature of Atherosclerosis
Source: Front Cell Dev Biol. 2022 Jan 5;9:800833. doi: 10.3389/fcell.2021.800833 (PMC8766414; doi:10.3389/fcell.2021.800833)
Supplement: Supplementary file 1 [file DataSheet1.docx]

Supplementary Material

## Supplementary Figures


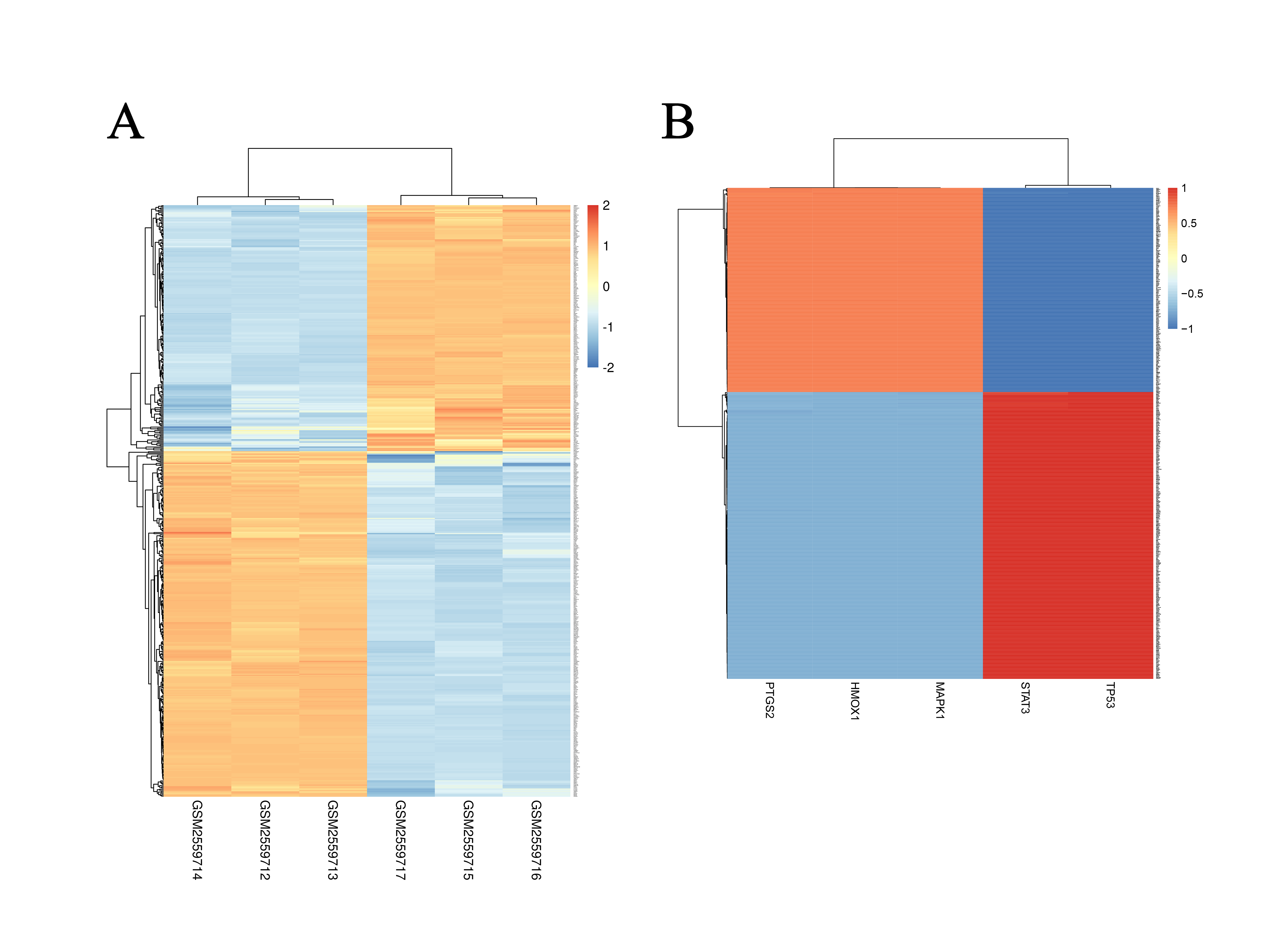


**Supplementary Figure 1.** Investigation of the relationship between the hub genes and DE-MFRGs. (A) Heatmap displayed the expression of the DE-MFRGs. (B) Heatmap indicating the correlation between the hub genes and DE-MFRGs.


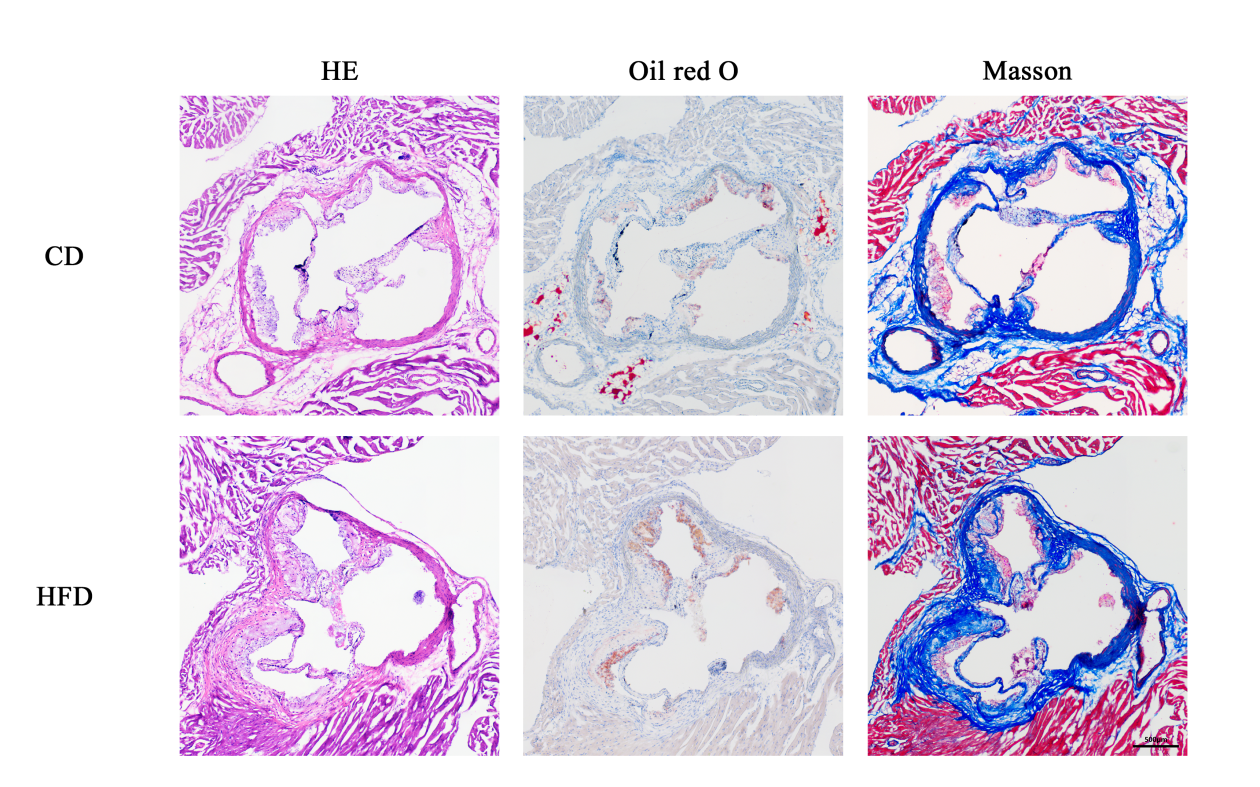


**Supplementary Figure 2.** Histological staining of aortic valve in *ApoE^-/-^* mice. Hematoxylin and eosin (HE), oil red O and Masson staining of atherosclerotic plaques in *ApoE^-/-^* mice.
